# Use of antagonists and morpholinos in loss-of-function analyses: estrogen receptor ESR2a mediates the effects of 17alpha-ethinylestradiol on primordial germ cell distribution in zebrafish

**DOI:** 10.1186/1477-7827-12-40

**Published:** 2014-05-15

**Authors:** Jingying Hu, Shaoyang Sun, Meng Guo, Houyan Song

**Affiliations:** 1 Department of Biochemistry and Molecular Biology, Shanghai Medical School and Key Laboratory of Molecular Medicine, Ministry of Education, Fudan University, Shanghai 200032, PR China

**Keywords:** 17alpha-ethinylestradiol, Primordial germ cells, Estrogen receptor

## Abstract

**Background:**

Various chemicals released into the aquatic environment adversely affect the reproductive system of fish, particularly by changing gonad structure and function. 17alpha-ethinylestradiol (EE2) is a potent environmental estrogen that disrupts sexual differentiation and normal reproduction in fish. Previous studies have shown that exposure to endocrine-disrupting chemicals (EDCs) disrupts the migration of primordial germ cells (PGCs) in zebrafish.

**Methods:**

To investigate the effects of EE2 exposure on PGC migration, zebrafish embryos were injected with *gfp-nanos* mRNA to label PGCs and subsequently exposed to different concentrations of EE2. Typical estrogen receptor antagonist treatment and morpholino knockdown experiments were used to identify functional estrogen receptors that mediate the effects of EE2.

**Results:**

The migration of PGCs was disrupted after exposure to high concentrations of EE2 (1 mirog/L). Loss-of-function analyses were performed for estrogen receptor ESR1, ESR2a, and ESR2b, and only loss of ESR2a resulted in a decreased number of ectopic PGCs following exposure to 1 mirog/L EE2.

**Conclusions:**

EE2 exposure disrupts PGC migration and distribution, and this effect is mediated through the estrogen receptor ESR2a.

## Background

The gonads are the primary targets of environmental pollutant toxicity. The mechanisms underlying defective gonadal development after exposure to pollutants have not been fully elucidated, and primordial germ cells (PGCs) may be the primary targets [1]. PGCs are progenitors of germ cells that migrate to the location of gonad development within 24 hours post fertilization (hpf). Recent studies have shown that *nanos* gene expression is a definitive marker of primordial germ cells in early zebrafish embryos [2,3]. To visualize PGCs, the coding sequence of green fluorescent protein (GFP) was fused to the 3′ un-translated region (3′UTR) of zebrafish *nanos1* mRNA, and mRNA transcribed from this construct was subsequently injected into zebrafish embryos [2].

17alpha-ethinylestradiol (EE2) is a potent environmental estrogen that has been shown to disrupt sexual differentiation and reproduction. The effects of EE2 are mediated through the transcriptional activities of the nuclear estrogen receptors, ESR1 and ESR2. Upon binding to a ligand in the nucleus, ESR1 and ESR2 bind to a specific estrogen response element (ERE) in the promoters of target genes. Zebrafish have a single *esr1* gene and two *esr2* genes, which encode ESR1, ESR2a, and ESR2b, respectively. Menuet et al. [4] showed differential regulation of ESR1, ESR2a, and ESR2b after exposure to estradiol-17beta. ICI, an estrogen receptor antagonist (ER-antagonist), blocks estrogen activity through two ER subtypes, ESR1 and ESR2 [5], and shows little selectivity in its activation of these receptors. Sun et al. [6] identified the estrogen receptor antagonist methyl-piperidino-pyrazole (MPP), which is ESR1-selective. Subsequently, Compton et al. [7] identified the potent and efficient ESR2 antagonist pyrazolo [1,5-a]pyrimidine to 2- phenyl −3- (4-hydroxyphenyl) -5,7- bis (trifluoromethyl) -pyrazolo [1,5-a] pyrimidine (PHTPP), which has minimal effects on ESR1.

In the present study, we examined the effects of EE2 exposure on the distribution of primordial germ cells in zebrafish embryos and characterized the roles of each estrogen receptor during this process. Exposure to 1 μg/L EE2 adversely affected the primordial germ cell distribution prior to gonad formation, and ESR2a played an important role in this process. These results may provide insight into the gonadal abnormalities observed in previous studies.

## Methods

### Zebrafish strain and maintenance

Wild-type zebrafish (AB* strain) were obtained from the Zebrafish International Resource Center (ZIRC, Oregon, USA). Embryos were collected following natural spawning. Wild-type zebrafish were raised, maintained, and staged as previously described [8]. In some cases, embryos and larvae were initially raised in water containing 0.2 mM 1-phenyl-2-thio-urea (PTU) to prevent pigment formation.

### Plasmid constructs

The *gfp-nanos*-*3′UTR* construct contained the GFP ORF fused to the 3′UTR of *nanos*. The 3′UTR of *nanos* was cloned from zebrafish cDNA using specific primers (Table 1). The GFP ORF was cloned from the vector pEGFP-1 (BD Biosciences Clontech, USA) using specific primers (Table 1). The amplified fragments were cloned into the pGEM-T vector (Promega, USA) using the restriction enzyme SacII.

**Table 1:** All primers used in this article

	**Primers**	
	**Sense**	**Antisense**
*nanos*-3′UTR	5′-GGAATTCAAAGCGCACACCAAGAGATT-3′	5′-TCCCCGCGGAATGTTTATATTTTCCTCACATTTTTC-3′
pEGFP-N1	5′-ATGGTGAGCAAGGGCGAGGA-3′	5′-TTACTTGTACAGCTCGTCCA-3′
*esr2a*	5′-GAAGATCTCACTGAGGAGTATCGAGGAC-3′	5′-TTGGATCCCAGAGCGGGACTGTAAAA-3′
*esr2b*	5′-GAAGATCTAGTTGGGCCTGAGATGCA-3′	5′-TTGGATCCTTAGGGCTCCGTGGTTGA-3′

pEGFP-N1-*esr2a* is a construct fusing the 5′UTR region of the *esr2a* gene to GFP to act as a reporter for morpholino knockdown effectiveness, so is pEGFP-N1-*esr2b. pEGFP-N1*-*esr2a* and *pEGFP-N1*-*esr2b* were constructed using specific primers listed in Table 1. The primers were designed based on the 5′-terminal sequence surrounding the putative start codons of zebrafish *esr2a* [Ensembl Transcript ID: ENSDART00000131069] and *esr2b* [Ensembl Transcript ID: ENSDART00000131800]. In total, 299 bp of the 5′UTR region of the *esr2a* gene and 256 bp of the 5′UTR region of the *esr2b* gene were PCR-amplified and cloned into the pEGFP-N1 vector (BD Biosciences Clontech, USA) at the BglI and BamHI restriction enzyme sites.

### Microinjection

For injection, mRNA was prepared using the mMessage mMachine kit (Ambion, USA). RNA was diluted in 10 mM HEPES (pH 7.6) and microinjected into zebrafish embryos at the one-to-four cell stage (200–400 pg/embryo).

Morpholino (MO) antisense oligonucleotides targeting the 5′UTR region of each gene were obtained from Gene Tools, LLC (USA). The following MO sequences were used: *esr2a*-MO: 5′-TGTCTCCTTCGGGATACTCGGACAT-3′ and *esr2b*-MO: 5′-AGCTCATGCTGGAGAACACAAGAGA-3′. One- and two-cell-stage wild-type zebrafish embryos were injected with MOs (2–5 ng/embryo).

The *pEGFP-N1-esr2a* and *pEGFP-N1-esr2b* plasmid constructs were prepared using a miniprep kit (Qiagen, USA). The plasmids were injected into one-cell-stage embryos at 5 ng/embryo (stock concentration of 50 μg/L DNA in 100 mM KCl containing 0.02% phenol red).

### Microscopy and imaging

The embryos and larvae were examined using an Olympus SZX12 microscope (Olympus, Japan), with or without a GFP filter, and photographed using a DP70 digital camera (Olympus, Japan).

### EE2 exposure and ER-antagonist co-exposure

Stock solutions of EE2 were prepared in 100% ethanol, and the exposure concentration were 1 ng/L, 10 ng/L, 100 ng/L, 500 ng/L, 1 μg/L and 2 μg/L. Stock solutions of ICI (ER antagonist), MPP (ESR1 antagonist), and PHTPP (ESR2 antagonist; Tocris, UK) were prepared in 100% ethanol. Each ER-antagonist was used at a concentration of 1 μg/L. Control fish were exposed to 1 μl/L ethanol. Every exposure group contained at least 100 chorionic embryos, and each experiment included four replicates for each treatment. After exposure for 24 h, the PGC distribution in each embryo was examined.

### Statistical analysis

The statistical software SPSS 18.0 was used to calculate the normality and homogeneity of variance of the data for embryos with ectopic PGCs. One-way analysis of variance (ANOVA) followed by Tukey’s test was used to identify differences in the percentage of embryos with ectopic PGCs among the various treatment conditions.

## Results

### Effects of EE2 on PGC distribution along the anterior-posterior axis

**Figure 1:**
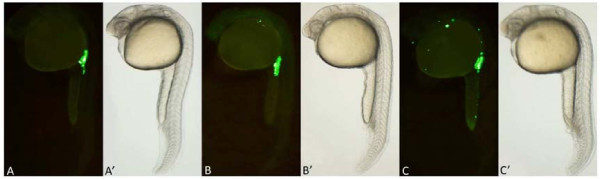
**Fluorescence images of zebrafish PGCs after exposure to various concentrations of EE2. A**: In all treatment groups, normal PGCs were observed in the anterior region of the yolk extension at 24 hpf. **B**: In the 1 ng/L, 10 ng/L, and 100 ng/L EE2 exposure groups, ectopic PGCs were primarily observed along the branchial arch, similarly to the spadetail mutant. **C**: In the 500 ng/L, 1 μg/L and 2 μg/L EE2 exposure groups, many ectopic PGCs were observed along the branchial arch, on the back, in the abdomen, and along the trunk. **A**’, **B**’, and **C**’: Bright-field views show the morphology of the embryos.

**Figure 2:**
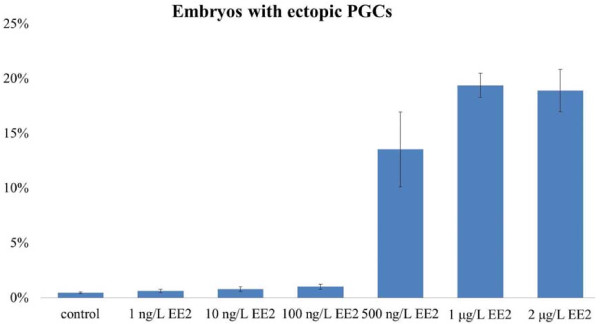
**Percentage of zebrafish embryos with ectopic PGCs in different exposure groups.** In the control, 1 ng/L, 10 ng/L, and 100 ng/L EE2 exposure groups, less than 2% of the zebrafish embryos showed ectopic PGCs. In the 500 ng/L EE2 exposure group, approximately 13% of zebrafish embryos contained ectopic PGCs. In the 1 μg/L EE2 exposure group, approximately 20% of zebrafish embryos contained ectopic PGCs. In the 2 μg/L EE2 exposure group, approximately 19% of zebrafish embryos contained ectopic PGCs. These rates were significantly higher than that of other groups (p < 0.01, one-way ANOVA followed by Tukey’s test).

We observed GFP expression in developing embryos after microinjection with *gfp-nanos*-3′UTR mRNA. GFP was expressed from the blastula stage in all injected embryos. At the 50% to 80% epiboly stage, a few cells showed stronger fluorescence than the surrounding cells, and these cells were referred to as brighter cells. The migration routes and final location of the brighter cells were similar to those described as PGCs in the zebrafish embryo [9,10]. To assess the impact of EE2 on PGC distribution, the embryos were injected with the *gfp-nanos*-3′UTR mRNA and were immediately exposed to different concentrations of EE2 till 24 hpf, when the cells are clustered along either side of the embryonic midline. At 24 hpf, PGCs were located below the somites and dorsal to the anterior end of the yolk extension in the non-EE2-treated group (Figure 1A). After 24 hours of EE2 treatment, the morphology of all embryos was normal, although some embryos displayed an abnormal PGC distribution pattern, in which ectopic PGCs were observed at the midbrain-hindbrain boundary and along the branchial arch at the 24 hpf stage, similar to the *spadetail* mutant [10] (Figure 1B). After exposure to 500 ng/L EE2, 13.5% of embryos displayed ectopic PGCs. In the 1 and 2 μg/L EE2 exposure groups, approximately 20% of the embryos showed ectopic PGCs. Ectopic PGCs were observed on the back or abdomen, or along the branchial arches of these embryos (Figure 1C). The percentage of ectopic PGCs in the EE2-treated groups was significantly higher than that in the other groups (p < 0.01, one-way ANOVA, followed by Tukey’s test) (Figure 2).

### Effects of estrogen receptor inhibition or knockdown on EE2-induced ectopic PGC distribution

To assess the involvement of estrogen receptors in the effect of EE2 on PGC distribution, typical estrogen receptor antagonists and specific estrogen receptor morpholinos were used. ICI was used to inhibit all ERs, MPP was used to inhibit ESR1, PHTPP was used to inhibit ESR2, and the *esr2a* and *esr2b* morpholinos were used to knock down the expression of ESR2a and ESR2b, respectively.

**Figure 3:**
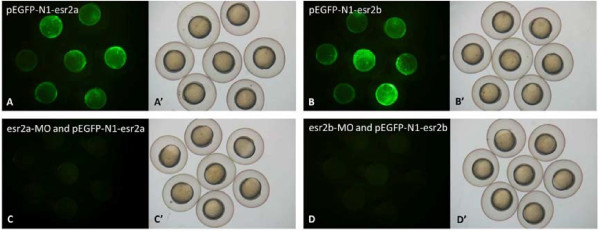
**Effects of MO on GFP expression in 7 hpf embryos injected with recombinant plasmids. A**, **B**: A mosaic pattern of GFP expression was detected throughout the embryos. **C**, **D**: GFP expression was undetectable after co-injection with MO and recombinant plasmids. **A**’, **B**’, **C**’, **D**’: Bright-field views show the morphology of the embryos.

To confirm the efficacy of the morpholino approach, *esr2a*-MO and *esr2b*-MO were co-injected with a green fluorescent protein (GFP) reporter containing the partial 5′UTR and start codons of *esr2a* and *esr2b*, separately. At 7 hpf, the *esr2a*-MO specifically knocked down the expression of GFP from the associated RNA transcript in 100% of embryos (n = 34) (Figure 3C). In contrast, GFP was highly expressed in the group of control embryos injected with only the plasmid (n = 29) (Figure 3A). These results confirmed the ability of the *esr2a*-MO to block the translation of the target protein sequence. Similar results were observed with the *esr2b*-MO (Figure 3B and D).

**Figure 4:**
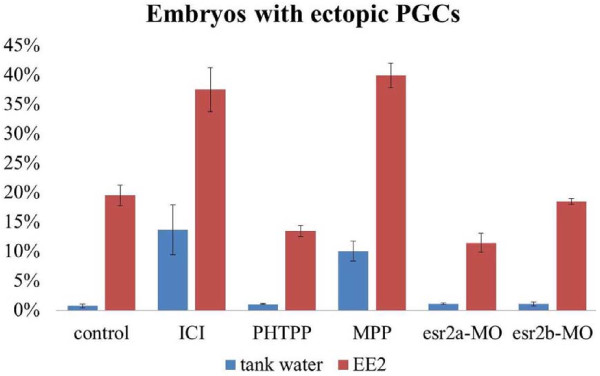
**Embryos with ectopic PGCs in different treatment groups.** In tank water, ICI and MPP treatment induced ectopic PGC distribution in 14% and 10% of the embryos, respectively. PHTPP, esr2a-MO, and esr2b-MO treatment did not disrupt PGC distribution. An increased percentage of fish with ectopic PGCs at 24 hpf was observed after exposure to EE2 compared with exposure to tank water alone (i.e., without EE2). Approximately 37% and 40% of the embryos displayed ectopic PGCs after exposure to ICI and MPP, respectively. Fewer embryos showed ectopic PGCs expression after exposure to PHTPP, and *esr2a* expression was reduced by approximately 13% and 11%, respectively. Inhibition of *esr2b* expression did not affect PGC distribution.

In most of the control non-EE2 treated embryos, PGCs arrived at their destination where the future gonad develops by 24 hpf (Figure 1). In contrast, ICI or MPP treatment disrupted PGC distribution in 14% and 10% of zebrafish embryos, respectively (Figure 4). Surprisingly, treatment with PHTPP, *esr2a*-MO or *esr2b*-MO alone did not disrupt PGC distribution (Figure 4). These results suggest that instead of ESR2, only ESR1 is required for normal PGC development.

In all of the 1 μg/L EE2 treated embryos, the gross morphology are normal. However, 1 μg/L EE2 treatment alone resulted in ectopic PGC in 19% of embryos. Compared to the 1 μg/L EE2 group, approximately 37% and 40% embryos showed ectopic PGCs after co-exposure to 1 μg/L EE2 and 1 μg/L ICI or 1 μg/L EE2 and 1 μg/L MPP, respectively. Addition of PHTPP or *esr2a*-MO in 1 μg/L EE2-treated groups resulted in fewer embryos with ectopic PGCs (approximately 13% and 11%, respectively), suggesting the adverse effect of EE2 on PGC distribution is mediated through ESR2a. Interestingly, injection of *esr2b*-MO in 1 μg/L EE2-treated embryos has no effect on PGC distribution compared to EE2 treatment alone, as shown by the number of embryos with ectopic PGCs are similar between the two groups. This result indicates that ESR2b does not mediate the effect of EE2 on PGC distribution (Figure 4).

## Discussion

In 2001, Koprunner et al. [2] cloned and identified the *nanos* gene as a marker of PGCs. The *nanos* mRNA is maternally loaded and plays an important role in PGC survival and migration. In the 3′UTR of *nanos* mRNA, a conserved sequence protects the mRNA from degradation in the PGCs [11]. In the present study, we fused the GFP coding region to the *nanos*-3′UTR. The translated GFP protein labeled PGCs with green fluorescence, as previously observed in somatic cells [3,12].

Previous studies have shown that environmental endocrine disruptors and estrogens affect the development and migration of fish PGCs and the development of gonads, thereby affecting reproductive function [1,13,14]. In the present study, we demonstrated that EE2 treatment disrupts PGC distribution in zebrafish and that ESR2a might play a role in this effect. EE2 exposure interferes with PGC distribution at a concentration range of 100–500 ng/L. Notably, higher doses of EE2 (>1 μg/L) did not produce stronger phenotypes.

PGC migration in zebrafish is well understood, particularly because guidance by the chemokine SDF-1a [15] and its receptor CXCR4b [15,16] has been characterized. CXCR7 is another receptor for SDF-1 [17,18] that promotes cell migration [17]. CXCR7 is crucial for proper migration of PGCs towards targets in the somatic environment rather than within migrating cells [19]. SDF1/CXCR4/CXCR7 signaling also participates in the migration of the posterior lateral line primordium, generating the embryonic posterior lateral line in zebrafish [20,21]. The estrogen receptor ESR1 represses the chemokine receptor CXCR4 and thus controls cell migration in the zebrafish posterior lateral line system [22]. Both the inactivation and overexpression of ESR1 result in aborted migration, confirming the importance of this receptor in SDF1-guided migration [22]. The synergistic effects of ICI or MPP and EE2 may explain the increased percentage of zebrafish with ectopic PGCs after ESR1 and all ERs were inhibited. Importantly, knocking down *esr2a* reduced the percentage of embryos with ectopic PGCs after exposure to high concentration of EE2, suggesting ESR2a may mediate the effects of EE2 on PGC migration. PHTPP inhibited ESR2, including both ESR2a and ESR2b, and this effect may have contributed to the weaker phenotype observed after co-exposure of PHTPP and EE2.

Functional gonad formation depends on the migration of PGCs from specification sites to locations of future gonad development [23-25]. This migration requires a chemokine micro-environment in which SDF-1a is secreted from somatic cells. The effects of EE2 on zebrafish PGC migration may reflect either changes in the PGC response to chemokines or interference of the micro-environment in which PGC migration occurs. Thus, the effects of EE2 on zebrafish PGC migration may be mediated by ESR2a, but the specific expression of ESR2a in PGCs or somatic cells as EE2 target sites remains to be determined.

## Conclusions

The present study showed that the migration and distribution of PGCs is affected after exposure to high doses of EE2 (500 ng/L), and approximately 20% of embryos displayed ectopic PGCs. Knocking down *esr2a* or application of a ESR2 inhibitor PHTPP, reduced the percentage of embryos with ectopic PGCs significantly after exposure to high doses of EE2, indicating that ESR2a may play an important role in EE2-related PGC phenotypes.

## Abbreviations

EDCs: Endocrine disrupting chemicals; EE2: 17α-ethinylestradiol; PGCs: Primordial germ cells; hpf: Hours post fertilization; GFP: Green fluorescent protein; 3′ UTR: 3′ un-translated region; ERE: Estrogen response element; ER: Estrogen receptor; MPP: Methyl-piperidino-pyrazole; PHTPP: Pyrazolo [1,5-a]pyrimidine to 2- phenyl −3- (4-hydroxyphenyl) -5,7- bis (trifluoromethyl) -pyrazolo [1,5-a] pyrimidine; PTU: 1-phenyl-2-thio-urea; MO: Morpholino; ANOVA: A one-way analysis of variance.

## Competing interests

The authors declare that they have no competing interests.

## Authors’ contributions

JYH participated in the design of the study, and drafted the manuscript. JYH and SYS conducted the data collection. JYH and MG participated in the statistical analysis, and performed the diagramming. HYS conceived of the study, participated in its design and coordination, and helped to draft the manuscript. All the authors read and approved the final manuscript.
